# Centenarian athletes: The paradigm of healthy longevity?

**DOI:** 10.1016/j.jnha.2025.100665

**Published:** 2025-08-29

**Authors:** Ángel Buendía-Romero, Enrique Higueras-Liébana, Luis M. Alegre, Ignacio Ara, Pedro L. Valenzuela

**Affiliations:** a GENUD Toledo Research Group, Faculty of Sport Sciences, University of Castilla-La Mancha, Toledo, Spain; b Centro de Investigación Biomédica en Red Fragilidad y Envejecimiento Saludable (CIBERFES), Instituto de Salud Carlos III, Madrid, Spain; c Grupo Mixto de Fragilidad y Envejecimiento Exitoso UCLM-SESCAM (TEC2022-007), Instituto de Investigación Sanitaria de Castilla-La Mancha (IDISCAM), Junta de Comunidades de Castilla-La Mancha (JCCM), Toledo, Spain

Global population aging has accelerated in recent decades partly due to increases in lifespan, and older adults are the fastest-growing population segment. Indeed, the number of centenarians is expected to increase fourfold by 2050. Despite this increase in longevity, this demographic phenomenon is accompanied by a rise in the prevalence of physical dysfunction and age-related disorders, which reduces the odds of healthy aging. Therefore, efforts are needed to promothe healthy aging even at the most advanced ages.

Regular physical exercise has been proposed as a cornerstone for promoting longevity and healthy aging [1]. Interestingly, this seems to be true even among centenarians, who tend to present a lower disease burden and a delayed onset of multiple conditions. Although controversy exists about whether centenarians exhibit a favorable genetic predisposition, a healthy lifestyle and particularly regular physical exercise at advanced ages appears to be a critical condition for attaining extreme longevity [2]. Indeed, a recent study in more than 5222 individuals aged ∼94 years reported that those who performed regular exercise had 31% higher odds of becoming a centenarian [2].

The benefits of exercise at the most advanced ages are also supported by interesting case studies. On July14th 2025, Fauja Singh, recognized as the first centenarian to finish a marathon, passed away after being hit by a car at the age of 114 in his native village in India. After spending most of his life working as a farmer, Fauja Singh began running at the age of 89 and physical exercise became a cornerstone of his daily routine. Despite this late start, he managed to improved his performance by 18% between his first marathon race (6 h 54 min in the 2001 London Marathon when he was 90 years old) and his personal record at the age of 92 (5 h 40 min in the 2003 Toronto Waterfront Marathon). In 2011, Fauja Singh became the first centenarian to complete a marathon, finishing the Toronto Waterfront Marathon in 8 h, 11 min, and 6 seconds. Additionally, in the same year, he achieved several world age group records for distances ranging from 100 m to 5 km. Fauja Singh’s last documented performance suggests an estimated peak oxygen uptake (V̇O_2peak_) of at least ∼23 ml·kg^−1^·min^−1^ [3], which is comparable to normative values (50th percentile) observed in men aged 60–69 years. This finding is clinically relevant because V̇O_2peak_ is a predictor of all-cause mortality, with each increase of 3.5 ml·kg^−1^·min^−1^ conferring a 14% improvement in survival regardless of sex, race or age (including among very old adults aged above 80 years) [4].

The case of Fauja Singh endorses the notion that it is never too late to start exercising, and that atonishing exercise-induced improvements in physical fitness can be obtained even at the most advanced ages. This is further supported by other cases such as that of Robert Marchand, a French centenarian who in 2012 set the world record for 1-h indoor (velodrome) cycling in the over-100 age group [5]. Even more surprisingly, two years later, during which he trained 5,000 km per year, he set a new record, and this considerable improvement in performance (+11%) was paralleled by a no less remarkable increase in V̇O_2peak_ (+13%) [5].

These feats must be viewed in the context of today’s aging societies. Currently, no drug can simultaneously improve all aspects of physiological function that determine physical independence which inevitably decline with age; however, exercise can have a multisystem rejuvenating effect. Indeed, physical exercise is currently one of the most effective interventions for counteracting age-related declines in cardiorespiratory function, cardiovascular health, as well as to prevent conditions such as sarcopenia (Fig. 1) [1]. Reaching 100 years of age with the physical function of these athletes can likely be considered the paradigm of healthy aging, with an active lifestyle (not only genetics) playing a major role in this process. In honor of Fauja Singh, who attributed his remarkable health to a simple lifestyle mantra: ‘*Eat less, run more, and stay happy — that is the secret behind my longevity*.’

**Fig. 1:**
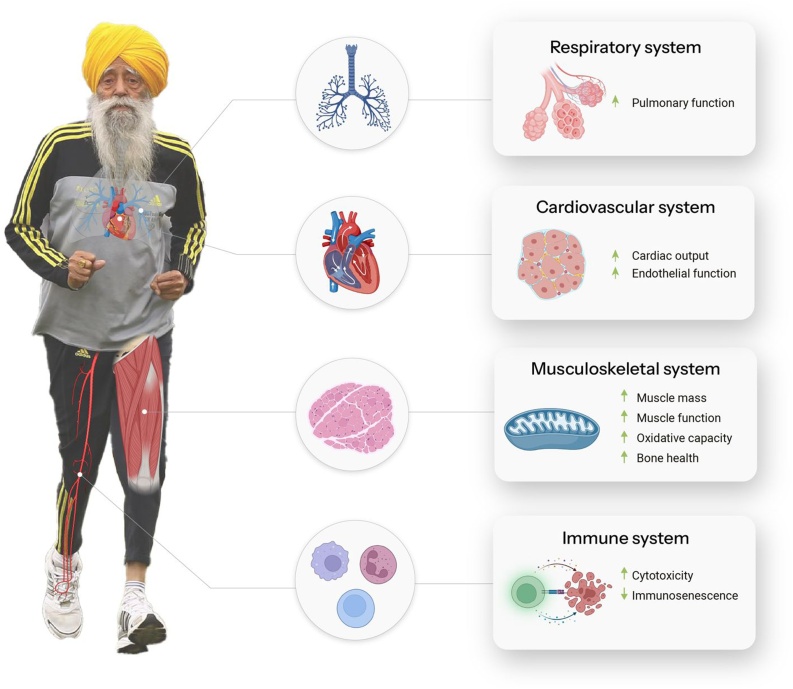
Summary of the benefits of regular physical exercise at the multisystem level during aging.

## Funding

All authors are supported by CIBERFES -Consorcio Centro de Investigación Biomédica en Red Fragilidad y Envejecimiento Saludable- (CB16/10/00477), Instituto de Salud Carlos III, Ministerio de Ciencia, Innovación y Universidades (Spain) and the Plan Propio de Investigación of the University of Castilla-La Mancha and FEDER funds from the European Union (2022-GRIN-34296). Á.B.-R. is supported by a postdoctoral contract granted by 10.13039/501100004837Spanish Ministry of Science and Innovation (JDC2023-052593-I, funded by MCIU/AEI/10.13039/501100011033). P.L.V is supported by a postdoctoral contract granted by University of Castilla-La Mancha and Fondo Social Europeo Plus (FSE+) (2024-UNIVERS-12850).

## Declaration of competing interest

The authors declare no conflicts of interest.
